# LAG-3 Contribution to T Cell Downmodulation during Acute Respiratory Viral Infections

**DOI:** 10.3390/v15010147

**Published:** 2023-01-03

**Authors:** Linmar Rodríguez-Guilarte, Mario A. Ramírez, Catalina A. Andrade, Alexis M. Kalergis

**Affiliations:** 1 Millennium Institute of Immunology and Immunotherapy, Departamento de Genética Molecular y Microbiología, Facultad de Ciencias Biológicas, Pontificia Universidad Católica de Chile, Santiago 8331150, Chile; 2 Departamento de Endocrinología, Facultad de Medicina, Pontificia Universidad Católica de Chile, Santiago 8331150, Chile

**Keywords:** LAG-3, Ligand, T cell dysfunction, viral infections

## Abstract

LAG-3 is a type I transmembrane protein expressed on immune cells, such as activated T cells, and binds to MHC class II with high affinity. LAG-3 is an inhibitory receptor, and its multiple biological activities on T cell activation and effector functions play a regulatory role in the immune response. Immunotherapies directed at immune checkpoints, including LAG-3, have become a promising strategy for controlling malignant tumors and chronic viral diseases. Several studies have suggested an association between the expression of LAG-3 with an inadequate immune response during respiratory viral infections and the susceptibility to reinfections, which might be a consequence of the inhibition of T cell effector functions. However, important information relative to therapeutic potential during acute viral lower respiratory tract infections and the mechanism of action of the LAG-3 checkpoint remains to be characterized. In this article, we discuss the contribution of LAG-3 to the impairment of T cells during viral respiratory infections. Understanding the host immune response to respiratory infections is crucial for developing effective vaccines and therapies.

## 1. Introduction

Lymphocyte activation gene 3 (LAG-3) or CD223 is an inhibitory receptor highly expressed on T cells, which is essential for downregulating T cell function, controlling autoreactivity, and decreasing inflammation during chronic infection or cancer disease [1,2,3,4,5,6]. Furthermore, there are cases where LAG-3 is responsible for mediating T cell dysfunction, known as cell exhaustion or depletion, which are T cells characterized by a progressive loss of effector functions that can prevent optimal control of infections and tumors [2]. T cell dysfunction has also been reported during acute infections, including respiratory viral infections [7,8,9,10]. These exhausted T cells lack early Interleukin-2 (IL-2) production capacity and display reduced proliferation, followed by defects in the production of Tumor necrosis factor-α (TNF-α), Interferon-γ (IFN-γ), and Granzyme B (GrzB) [1,2,3,4]. In addition, virus- or target-specific suppressed T cells show a characteristic phenotype expressing multiple inhibitory receptors, including LAG-3, Programmed cell death protein 1 (PD-1), T cell immunoglobulin and mucin-domain containing-3 (TIM-3), Cytotoxic T-lymphocyte antigen 4 (CTLA-4), T cell immunoglobulin, and immunoreceptor tyrosine-based inhibitory motif (ITIM) [3,4]. These inhibitory receptors regulate T cell responses by directly inhibiting effector T cell activation, promoting regulatory T cell suppressive function, or modulating antigen-presenting cell (APC) function to reduce T cell activation [11]. The expression of the inhibitory receptor repertoire changes according to the activation state of the T cell and the tissue microenvironment, thereby exerting tight control over the T cell response that is important for the functioning of the immune system [1,2,3,4].

Continuous exposure of the human respiratory tract to the environment makes it more susceptible to viral infections [12]. The severity of the illness associated with respiratory viral infections depends on several factors, including immunomodulation, which is crucial for virus containment [12]. During the course of a viral infection, innate immunity triggers pro-inflammatory responses in the early stage of infection, and adaptive immunity has a critical role in eliminating viral pathogens during the later stages of infection [13,14]. Interestingly, during the last phase of infection, the T cells regulate the inflammatory response and promote the development of immunological memory [15]. The viral clearance during this stage is mediated by CD8^+^ T cell subsets, which might be activated in a CD4^+^ T cell-dependent or independent manner [16,17,18]. Therefore, the correct functioning of CD8^+^ T cells is essential for the elimination of pathogenic viruses during an infection.

CD8^+^ T cells exert their effector function by eliminating intracellular pathogens by releasing perforin and GrzB that lyse infected cells or by upregulating molecules that induce cellular death [18,19,20]. Once a respiratory virus is cleared, normal pulmonary homeostasis must be restored [21]. Downmodulation of T cell function likely represents a regulatory mechanism to restore the normal pulmonary state by reducing the activity or survival of virus-specific cytotoxic CD8^+^ T cells [21,22]. However, T cell-mediated inflammation has also been shown to contribute to lung damage and clinical disease during acute respiratory infections, such as human metapneumovirus (hMPV), human respiratory syncytial virus (hRSV), influenza virus, and severe acute respiratory syndrome coronavirus 2 (SARS-CoV-2) [23,24,25,26,27]. These are the most prevalent respiratory viruses that cause acute lower respiratory tract infections (ALRTI); they affect millions of people of different age groups annually, especially infants, young children, the elderly, and immunocompromised patients [28,29]. Therefore, knowing how these respiratory viruses modulate the CD8^+^ T cells is essential.

Additionally, virus-specific memory CD8^+^ T cells can protect against a secondary viral infection by reducing viral lung loads and killing virus-infected cells [15,27,30]. Interestingly, LAG-3 plays an essential role as a negative regulator of CD8^+^ T cell function by suppressing cytotoxic activity [31]. However, a sustained expression of LAG-3 can affect the development of memory responses [7,32]. The failure to generate a quality memory CD8^+^ T cell response, which is caused by the continuous expression of LAG-3, might explain the ability of respiratory viruses to reinfect people despite minimal antigenic drift [7,8,32,33]. Despite this complexity, the functional synergy that PD-1 and LAG-3 receptors usually exert can be used to restore immune functions [7]. Immunotherapy directed at immune checkpoints, such as LAG-3, has arisen as a promising strategy for controlling malignant tumors and chronic viral diseases [34]. However, important information relative to the therapeutic potential during ALRTI and the mechanism of action for these checkpoint receptors remains to be characterized [3,4]. In this article, we discuss in detail the current literature that describes the biology of LAG-3, the relevance of this inhibitory receptor in T cell immunomodulation during ALRTIs caused by the most prevalent respiratory viruses, and the therapeutic potential of this molecule during viral infections.

## 2. General Characteristics of LAG-3

LAG-3 is a type I transmembrane protein that was first identified in 1990, and is expressed on the surface of various immune cells, including T cells [35]. This receptor can regulate T cell activity by inhibiting the activation and effector functions of these cells [6]. LAG-3 activation promotes a suppressive immune response, reducing cytokine and granzyme production in effector T cells and promoting differentiation of regulatory T cells (Tregs) [36,37,38,39]. In this section, we will further describe the characteristics of the LAG-3 receptor in T cells.

### 2.1. Molecular Organization

**Figure 1:**
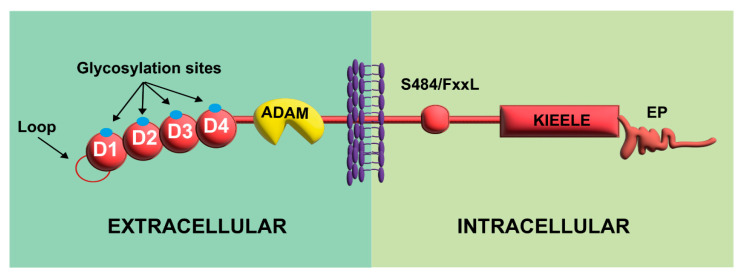
Extracellular and intracellular structure of LAG-3. The receptor LAG-3 comprises four extracellular domains, D1–D4, with D4 located closer to the extracellular membrane and D1 more distal. Additionally, the extracellular domains possess multiple glycosylation sites and a loop in D1 that promotes interaction with MHC-II. An extracellular domain can undergo proteolytic cleavage by ADAM. The intracellular domain contains a serine phosphorylation site (S484/FxxL motif), a unique amino acid sequence (KIEELE), and a glutamic acid and EP repeat.

The LAG-3 gene includes eight exons, encoding a transmembrane protein of 498 amino acids [35]. LAG-3 possesses four immunoglobulin (Ig)-like domains (D1 to D4) in the extracellular region with multiple glycosylation sites (Figure 1) [35]. In addition, LAG-3 possesses two unique structural features in the extracellular region [35,40]. First, a proline-rich amino acid loop within D1 allows LAG-3 dimerization and mediates the interaction between LAG-3 and Major Histocompatibility Complex Class II (MHC-II). This likely disrupts the interaction of the glycoprotein called cluster of differentiation 4 (CD4) with MHC-II [40]. Second, LAG-3 is more susceptible to a disintegrin and metalloproteinase (ADAM) shedding since it has a longer connecting peptide between D4 and the transmembrane region, allowing the formation of a soluble ligand (sLAG-3) [40,41].

The cytoplasmic tail of LAG-3 has three conserved motifs (Figure 1) [42,43,44]. The first contains a putative serine phosphorylation site (S484/FxxL motif), and no specific function has been attributed to it apart from its negative association with IL-2 production [42]. The second motif, KIEELE, corresponds to a highly conserved short sequence not found in other proteins, which is required for LAG-3 to downregulate T cell function [43]. However, KIEELE-independent intrinsic mechanisms have been shown to mediate the inhibitory signal of LAG-3 in T cells [42]. The third motif is a proline dipeptide (EP motif) that is critical for LAG-3 trafficking to the cell surface after stimulation and for its colocalization with CD3/TCR, CD4, and CD8 (Figure 1) [44]. After clarifying the structure of LAG-3, it is necessary to know the ligands for this receptor and the inhibition mechanisms of activated T cells that will be discussed below.

### 2.2. Expression of LAG-3 and Its Ligands

LAG-3 and its ligands are expressed in various immune cells, such as T cells, B cells, and dendritic cells (DCs) [37,45,46]. Several characteristics of this receptor have been described in different cell types. In T cells, this receptor negatively regulates homeostatic activation, proliferation, and expansion [47,48]. LAG-3 has also been identified as a marker for a subset of IL-10-producing Treg cells [37]. The expression of LAG-3 on Treg cells induces their suppressive function, reducing the proliferative capacity of effector cells [37]. On the other hand, in B cells, LAG-3 expression is dependent on T cells and plays a regulatory role [46]. A subset of LAG-3^+^ regulatory B cells has been described in plasma cells that produce IL-10, suppressing B cell activity [49]. LAG-3 is also expressed in different subtypes of DCs, contributing to immune homeostasis regulation and maturation of DCs [47,50].

**Figure 2:**
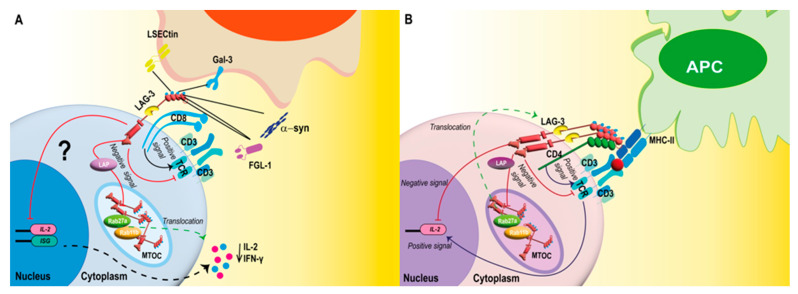
LAG-3 binds to various ligands activating an inhibitory signaling pathway in T cells. LAG-3 colocalizes in the membrane with the CD3/TCR complex and CD8 (**A**) or CD4 co-receptors (**B**). In CD8^+^ T cells, LAG-3 recognition occurs by alternative ligands (Gal-3, FGL-1, LSECtin, and α-syn). Gal-3 and LSECtin bind to LAG-3 through glycosylation sites, whereas FGL-1 binds to LAG-3 through the D1 and D2 domains, and α-syn binds to LAG-3 through the D1 domain (**A**). In these cells, LAG-3 downregulates the TCR leading to decrease secretion of IL-2 and IFN-γ (**A**). In CD4^+^ T cells, LAG-3 binds to its primary ligand, MHC-II. After the immunological synapse takes place, the CD4 co-receptors send positive signals to the nucleus that lead to the activation of *Il-2* and the expression of this cytokine (**B**). In contrast, LAG-3 sends negative signals through its cytoplasmic domains. In both cases (**A**,**B**), the EP motifs recruit the LAP protein, which mediates LAG-3 transport and colocalization to the cell surface, enhancing the dimerization/oligomerization of LAG-3. This process is necessary for MHC-II/LAG-3 binding (**B**). The KIEELE cytoplasmic domain of LAG-3 is responsible for the inhibition of the first steps of the TCR pathway. KIEELE possesses a lysine residue that prevents the activation of transcription factors, including the IL-2 transcription factor (**B**).

MHC-II molecules, transmembrane αβ heterodimer antigen-presenting proteins expressed on APCs, are the primary canonical ligand binding to LAG-3 with a high affinity [39,51,52]. MHC-II binds to the loop of the D1 domain of LAG-3 and transduces inhibitory signals through its cytoplasmic domain (Table 1) [52]. Four ligands have been described other than MHC-II: Galectin-3 (Gal-3), Fibrinogen-like protein 1 (FGL-1), Liver Sinusoidal Endothelial Cell lectin (LSECtin), and α-synuclein (α-syn) [53,54]. Gal-3 is a 31 kDa β-galactoside-binding lectin expressed in different cells, such as neutrophils, macrophages, mast cells, and sensory neurons in several tissues, including the lung epithelium and endothelium (Table 1) [55,56]. Gal-3 interacts with LAG-3 through the glycosylation sites on the extracellular domain, thus exerting its regulatory function on CD8^+^ T cells [55]. This interaction downregulates T cell proliferation and their ability to produce IL-2 and IFN-γ (Figure 2A) [55]. FGL-1 is a 68 kDa protein member of the fibrinogen-associated protein 1 (FREP) family and contains two homodimers connected by disulfide bonds; it is expressed by some neoplastic cells and secreted by hepatocytes (Table 1) [57,58]. The interaction occurs by binding D1 and D2 domains of LAG-3 with FGL-1, leading to reduced IL-2 levels in the tumor microenvironment (Figure 2A) [54]. LSECtin is a type II integral membrane protein of approximately 40 kDa that regulates CD8^+^ T cell function in tumor environments by inhibiting their cytotoxic function [59]. LSECtin is expressed in liver sinusoidal endothelial cells, endothelial cells of the lymph nodes, and melanoma cells [59,60]. This molecule is a lectin with carbohydrate-recognition domains that may bind to glycosylated sites of LAG-3 (Table 1) [61]. This interaction with LAG-3 inhibits the secretion of IFN-γ in effector T cells (Figure 2A) [60]. Lastly, α-syn is a neuronal protein 140 amino acids long in presynaptic nerve terminals [62]. In patients with Parkinson’s disease and other neurodegenerative disorders, preformed fibrils (PFF) of α-syn could preferentially bind to the D1 domain of LAG-3 with high affinity, promoting the loss of dopaminergic neurons and behavioral deficits (Table 1) [63].

**Table 1:** LAG-3 binding ligands distribution and function.

Ligand	LAG-3 Binding Site	Cell and Tissue Distribution	Function	Reference
MHC-II	Loop of the D1 domain.	APCs (macrophages, DCs, and B cells).	Disrupt the interaction of CD4^+^ with MHC-II.	[39,52]
Gal-3	Glycosylation sites.	Macrophages, monocytes, DCs, eosinophils, mast cells, NK, activated T and B cells, epithelial cells, endothelial cells, and sensory neurons.	Inhibit the CD8^+^ T cell cytotoxic function.	[55,56]
FGL-1	D1 and D2 domains.	Neoplastic cells and hepatocytes.	Reduce IL-2 levels in the tumor microenvironment.	[54]
LSECtin	Glycosylation sites.	Liver sinusoidal endothelial cells, endothelial cells of the lymph nodes, and melanoma cells.	Inhibit the cytotoxic functions of NK and CD8^+^ T cells in tumors. Inhibit IFN-γ secretion in effector T cells.	[59,60,61]
α-syn	D1 domain.	Presynaptic nerve terminals.	Enhance the loss of dopaminergic neurons and behavioral deficits.	[63]

### 2.3. LAG-3 Signaling

T cell activation upon antigen recognition via the T cell receptor (TCR) is controlled by antigen-independent signals through stimulatory or inhibitory receptors that optimize immune responses [5,64,65]. LAG-3 colocalizes with other molecules, such as CD3, CD4, or CD8, in cholesterol-rich raft areas during the early steps of antigen recognition (Figure 2A,B) [5]. This colocalization allows the association of LAG-3 with CD3 in the TCR complex, as well as LAG-3 cross-linking during immunological synapse assembly, which downregulates signal transduction activity [5]. Moreover, in the cytoplasm, LAG-3 colocalizes with the endosomal recycling compartment protein (Rab11b) and the lysosomal secretory pathway marker (Rab27a), which could suggest that LAG-3 tends to be continuously recycled, facilitating a rapid translocation to the membrane by the pathway lysosomal secretory pathway through the microtubule-organizing center (MTOC) after T cell activation (Figure 2B) [66]. The amount of expression of LAG-3 on the cell surface is strongly associated with its inhibitory activity [42].

The cytoplasmic tail of the LAG-3 plays a critical role in the mechanisms of T cell function inhibition [42,43]. The KIEELE motif was initially identified as the crucial driver of LAG-3 signal transduction [43]. However, recent studies using an in vitro T cell activation system with LAG-3-MHC-II blocking mAbs suggested that the proximal region of the membrane containing the FxxL motif, including the residues F475 and L478, is the main responsible for inhibiting T cell activation by directly suppressing the secretion of IL-2 or the response to this cytokine (Figure 2B) [42]. The similarity between the FxxL motif and the YxxL motif, which are located in immunoreceptor tyrosine-based inhibitory motifs (ITIM), means that phosphotyrosine-independent signaling adaptor proteins might also be involved in LAG-3-induced inhibitory signaling [42]. Other inhibitory signals are associated with the recruitment and colocalization of LAG-3 on the cell surface and depend on the EP motif (Figure 2B) [42].

Interestingly, an intracellular factor for the EP motif was shown to recruit LAG-3-associated protein (LAP) directly, where LAP assembles LAG-3 into lipid rafts induced after TCR signaling (Figure 2B) [5]. LAP mediates LAG-3 transport and colocalization to the cell surface, which can enhance the dimerization/oligomerization of LAG-3, a process that is necessary for MHC-II/LAG-3 binding (Figure 2B), but not for the binding to other ligands [5,67]. Recent studies have described how, during the immunological synapse for CD4^+^ and CD8^+^ T cells, LAG-3 associates with the TCR-CD3 complex and interacts through the EP located in the cytoplasmic domain, leading to a pH reduction with a phosphorylation decrease on the tyrosine kinase ZAP70, which reduces T cell activation (Figure 2A,B) [68]. Even though the differential function elicited by LAG-3 on CD4^+^ and CD8^+^ T cells has not yet been the subject of many studies, it is necessary to know the mechanisms by which LAG-3 participates in the inhibition of CD8^+^ cells because these cells do not recognize the main LAG-3 ligand, MHC-II [69]. Diverse mechanisms have been proposed to study this differential function, such as identifying alternative LAG-3 ligands, including LSECtin, Gal-3, α-syn, and FGL-1 binding to LAG-3 in CD8^+^ T cells and the co-distribution of LAG-3 and CD8 coreceptor during early T cell activation events (Figure 2A) [70,71].

LAG-3 expression is regulated at the cell surface, partly by cleavage of the extracellular domain by TCR-induced disintegrins and metalloproteases (ADAM10 and ADAM17) [41]. Cleavage gives rise to a 52 kDa soluble form of sLAG-3 [72]; sLAG-3 mediates the activation of APCs, thus activating and promoting the production and proliferation of CD8^+^ T lymphocytes, favoring a cellular response characterized by a T_h_1 profile [73,74].

### 2.4. LAG-3 Mediates Immunometabolic Programming of T Cells

The metabolic programs of T cells match their functional demands [75]. A specific metabolic profile is necessary for T cells to maintain their effector functions and eliminate pathogens [76,77]. Naïve T cells predominantly rely on mitochondrial oxidative phosphorylation to generate ATP, whereas an activated T cell displays robust aerobic glycolysis to maximize macromolecule synthesis and energy [76]. Memory T cells revert to oxidative and fatty acid metabolism, resembling the metabolic phenotype of naïve T cells [76]. Interestingly, these metabolic processes can be regulated through cell signaling, gene transcription, and protein synthesis, as well as an inadequate supply of nutrients and oxygen or the accumulation of metabolic intermediaries [76]. In CD4^+^ T cells, LAG-3 maintains the metabolic and mitochondrial quiescence of naïve CD4^+^ T cells, limiting oxygen consumption and respiratory capacity and preventing excessive proliferation [78]. LAG-3 deficiency leads to the activation of the signal transducer and activator of transcription 5 (STAT5), leading to increased glycolytic capacity and effector function after activation [78]. Furthermore, upregulation of STAT5 expression reduces CD4^+^ T cell dependence on IL-7 for survival and metabolism [78]. Other evidence shows that metabolic reprogramming of diabetogenic CD4^+^ T cells using a competitive inhibitor of the limiting enzyme of glycolysis 6-phosphofructo-2-kinase/fructose-2,6-bisphosphatase 3 (PFKFB3) increases the expression of LAG-3 and PD-1 [79]. On the other hand, LAG-3 deficiency alters APC metabolism, and cell metabolism shifts from oxidative phosphorylation to energy production via the glycolytic pathway, altering APC cytokine secretion through an increase in TNF-α and a reduction in IL-10 production [80].

Currently, there is limited information on the metabolic consequences of LAG-3 on CD8^+^ T cells. However, the generation of long-term memory can be affected by the high glycolytic rate in T cells since the increase in glycolytic activity leads to a state of terminal differentiation in these cells [81]. In this sense, the expression of LAG-3 may play an essential role in CD8^+^ T cell memory responses by maintaining cellular metabolism in oxidative phosphorylation, as occurs with APC [80].

## 3. Respiratory Viral Infections Modify the Expression of LAG-3

As mentioned above, LAG-3 plays an essential role in negatively regulating the activation of effector CD8^+^ T cells [82]. In this line, respiratory viruses can modulate the expression of LAG-3 to their advantage to prevent the activation of CD8^+^ T cells [7]. The up-regulation of LAG-3 during viral infection may generate an incomplete cytotoxic response that impairs viral clearance and immunological memory [7,8,32,83,84]. This section will discuss the effects of increasing the expression of LAG-3 on CD8^+^ T cells during acute infections caused by the most prevalent respiratory viruses, such as SARS-CoV-2, influenza virus, hMPV, and hRSV.

### 3.1. Severe Acute Respiratory Syndrome Coronavirus 2

SARS-CoV-2 is the viral pathogen responsible for the 2019 pandemic and the etiological agent for Coronavirus disease 2019 (COVID-19) [85]. Since then, SARS-CoV-2 has become one of the most prevalent respiratory viruses, affecting people of all ages [86,87,88,89,90,91]. Patients infected with this virus commonly show symptoms that include fever, cough, and headache; in more severe cases, the patients can develop pneumonia and acute respiratory distress syndrome (ARDS) [90,91]. Additionally, SARS-CoV-2 infection can reach various organs, such as the kidney, heart, and brain, and cause damage to all of them [90,91].

Samples from severe SARS-CoV-2 patients have demonstrated an increase of innate immune cells, such as neutrophils and NK cells, while observing a downmodulation in T cells [85,90,92]. The reduced T cell response might be associated with the lack of long-lasting immunity that lymphocytes are usually responsible for achieving [85,90,92]. Other human lung tissue samples and nasopharyngeal lavage have shown an overexpression of inhibitory receptors on T cells, such as LAG-3 [9,93,94,95,96,97,98]. This observation could explain the significantly reduced numbers of T cells in severely ill patients. Severe COVID-19 patients have been reported to have reduced MHC-II expression in various APCs [99]. This reduction, accompanied by increased LAG3 expression on the surface of T cells, may cause an imbalance in the interactions between these two molecules. This may reflect a failure in the proper interaction between innate and adaptive cells and result in aberrant expression of cytotoxic cytokines [99]. This alteration could contribute to the immunopathology of SARS-CoV-2 [99].

Interestingly, patients with severe SARS-CoV-2-infection promote the expression of LAG-3 in T cells is regulated through the genes activated downstream of the IFN-I signaling [99,100]. An in vitro study performed with cells from critically ill COVID-19 patients with a predominant IFN-I response and T cells revealed two mutually antagonistic modules of ISG regulators, in which SP140 is a bidirectional regulator for LAG-3 and TIGIT under IFN-I responses [100]. The transcription factor SP140 promotes an increase in the expression of LAG-3 but decreases TIGIT expression in response to interferon [100]. Lymphopenia and expression of inhibitory receptors in the T cells of patients who are severely ill with SARS-CoV-2 suggest that the loss of function in these cells can worsen pathology by interfering with the eradication of the virus.

### 3.2. Influenza Virus

The influenza virus is a respiratory virus that causes considerable morbidity and mortality in humans every year, leading to symptoms such as fever, cough, and pneumonia in the more severe cases [29]. The cases with the highest rates of influenza-related mortality occur in the elderly and those with underlying medical conditions [101]. In addition, it has been observed that influenza virus infection produces excessive inflammation that can cause severe damage to the host [102].

Since LAG-3 plays a regulatory role in immune responses [83,84,103], it can be suggested that LAG-3 may have a role during the period of infection with the influenza virus. It has been shown that CD4^+^ T cells that recognize influenza virus hemagglutinin protein also express LAG-3 [84,104]. This observation suggests that recognition of this protein can induce the expression of LAG-3. Interestingly, T_h_1 cells with high expression of LAG-3 can suppress CD8^+^ T lymphocytes and restrict lung inflammation without affecting the clearance of this virus [83]. Furthermore, it has been observed that the blockade of LAG-3 promotes the activation of CD8^+^ T cells stimulated with peptides from the influenza virus [7]. These results suggest that the influenza virus hemagglutinin might positively modulate the effect of LAG-3, leading to impairment of CD8^+^ T cells activation. However, further studies are required to understand the mechanism behind this modulation over LAG-3.

### 3.3. Human Metapneumovirus

An important cause of ALRTI in infants, hMPV is the second most frequently identified pathogen after hRSV in lower respiratory diseases, including bronchiolitis, pneumonia, and croup [105,106,107]. One distinctive aspect of this virus is that it can cause multiple reinfections throughout the infant’s life [108,109].

*In vivo* studies have shown that the depletion of CD8^+^ T cells may increase reinfections cases that are caused by hMPV [7]. These cells lose the ability to secrete the cytokines necessary to eliminate hMPV [7,8,33]. In addition, increased LAG-3 expression has been described in pulmonary CD8^+^ T cells that leads to mouse susceptibility to hMPV reinfection [32]. Interestingly, LAG-3 blockade can restore cytokine production, suggesting the crucial involvement of LAG-3 during the infection with hMPV [7]. Furthermore, experiments with PD-1 deficient mice demonstrated that using LAG-3 blockade during infection with hMPV can restore CD8^+^ T cell function, but increases pulmonary pathology [7]. These studies suggest that, during the infection with hMPV, the expression of LAG-3 increases in pulmonary CD8^+^ T cells to control the inflammatory response elicited against the virus, displaying a protective role during this infection.

### 3.4. Human Respiratory Syncytial Virus

In children under two years of age, hRSV is the principal cause of ALRTI, leading to cough, fever, wheezing, bronchiolitis, and pneumonia in more severe cases [110,111,112,113,114]. The hRSV belongs to the *Pneumoviridae* family, the same family as the hMPV [115]. Even though there is no information about the expression of LAG-3 during hRSV infection, a role could be suggested based on what is known for hMPV. As mentioned previously, hMPV infection causes an increase in LAG-3 expression, possibly as a protective regulatory response [7,8,33]. Based on this information, it could be suggested that during infection with hRSV, the expression of LAG-3 also increases to inhibit the activation of CD8^+^ T cells. However, the response of CD8^+^ T cells is robust during hRSV infection [116,117], which indicates that LAG-3 possibly behaves differently during the hRSV infection, decreasing the activity of LAG-3.

## 4. Therapeutic Perspectives Targeting LAG-3

LAG-3 has been described as an essential next-generation immune checkpoint receptor proposed as a therapeutic target against cancer, autoimmune and inflammatory diseases, parasitic infections, and acute and chronic viral infections [10,118,119,120,121]. Research on the use of the LAG-3 receptor has focused on immunotherapies, especially for treating different types of cancer [119,122]. Several formulations have been explored, including soluble dimeric LAG-3 as an adjuvant, agonist antibodies that block the interaction between this receptor and its ligands, antibody-mediated LAG-3 cell depletion in autoimmunity, and finally, the use of small molecule GSK-3 to modulate LAG-3 expression [123]. Clinical trials have shown that LAG-3 blockade as a cancer treatment activates APC to promote DCs proliferation and enhances regulatory T cell immunosuppression and antigen cross-presentation to CD8^+^ T cells, promoting an anti-tumor state [124]. Therefore, LAG-3 blockade has been used in these pathologies as monotherapy or in combination with other agonists, such as anti-PD1, showing better results [125]. However, it has also been described that using LAG-3 inhibitors can help overcome immune exhaustion during persistent inflammatory states, such as cancer and viral infections [126]. In this case, research has mainly focused on treating LAG-3-blocking antibodies during chronic viral infections. However, an in vivo study evaluated and compared the role of LAG-3 in both chronic and acute viral infections, resulting in a similar intrinsic cellular effect of LAG-3 in both cases, thus supporting the use of LAG-3 blockers to enhance CD8^+^ T cell responses [121]. However, it does not generate the same results under other circumstances, as demonstrated by a study in a mouse model infected with lymphocytic choriomeningitis virus, where LAG-3 blockade failed to either rescue CD8^+^ T cell cytokine production or to affect viral titers [127].

Similarly, in a PD-1-deficient mouse model, it was possible to observe that using an antibody against LAG-3 resulted in restoring CD8^+^ T cell effector functions [7,121,127]. However, it was also found that the restored effector function of CD8^+^ T cell increased the lung immunopathology when mice were infected with hMPV, suggesting that LAG-3 contributes to the protection against the development of the immunopathology during hMPV shedding [7,121,127]. Phase I clinical trials have highlighted the role of sLAG-3, known as IMP321, as an adjuvant in a trivalent influenza vaccine that demonstrated safety, tolerability, and immunogenicity [128]. In addition, IMP321 has been described to enhance the response of T_h_1 influenza-specific CD4^+^ cells by increasing the secretion of IFN-γ, the prototypical T_h_1 effector cytokine, TNF-α, an inflammatory cytokine, and IL-2, which is involved in the development of memory T cells [128]. More recently, based on studies showing a transient increase in the expression of this receptor during immune activation as part of COVID-19, the use of LAG-3 blockers (alone or in combination with other checkpoint inhibitors) during the early or late phase of SARS-CoV-2 infection has been proposed as an effective therapeutic measure for treatment [9,97]. However, additional studies are required to support this strategy conclusively. Considering the current pandemic and the lack of treatments against other viruses such as hRSV or hMPV, exploring the use of inhibitors for this receptor as a therapy against acute respiratory diseases would be important.

## 5. Concluding Remarks

LAG-3 is an immune checkpoint receptor that can have beneficial effects, serving as an adjuvant for vaccines and immune treatments, contributing to the generation of T_h_1-type antiviral responses, and suppressing excessive cytotoxic activity. However, the role of LAG-3 in ALRTI is still controversial. In addition, LAG-3 can contribute to reducing an exacerbated inflammatory response and also quickly suppress the response of CD8^+^ T cells. CD8^+^ T cells play a fundamental role in viral eradication and the generation of immunological memory. LAG-3 expression can affect the function of these cells, which can interfere with viral eradication and prevent the generation of long-lasting memory responses. On the other hand, blockade or genetic ablation of LAG-3 improves viral eradication and could contribute to the generation of longer-lasting memory responses, implying a protective response against reinfections.

In contrast, the generation of a more robust immune response involves the release of pro-inflammatory cytokines such as TNF-α and IFN-γ, in addition to GrzB and Perforin, which can cause tissue damage and increase immunopathology. Based on these features, it is likely that LAG-3 expression could work as a biomarker to predict the outcome of acute infections. Its use as a marker for immune cell activation or inhibition during acute infections may be advantageous for considering treatment options. In addition, it is essential to evaluate the effect of LAG-3 targeted immunotherapy for treating malignancies or enhancing the effectiveness of vaccines and antiviral treatments. Studies designed to approach this question became important because treatment with anti-LAG-3 can alter humoral and cellular immune responses. Further studies are needed to evaluate the potential therapeutic implications of sLAG-3 or LAG-3 blockade in viral ALRTIs and their possible use as vaccine adjuvants.

## Data Availability

Not applicable.
